# Professional Values, Work Environment and Job Satisfaction Among Critical Care Nurses in Palestine: A Descriptive Cross‐Sectional Study

**DOI:** 10.1002/nop2.70722

**Published:** 2026-07-30

**Authors:** Bahaaeddin M. Hammad

**Affiliations:** ^1^ Faculty of Nursing Arab American University Jenin Palestine

**Keywords:** critical care nurses, intention to leave, job satisfaction, nursing work environment, Palestine, professional values, workload

## Abstract

**Background:**

Critical care nurses work in demanding clinical environments where professional values, staffing conditions, ethical climate, workload and burnout may influence job satisfaction. Understanding these relationships is important for supporting nurse retention and stable, safe intensive care practice.

**Aim:**

To assess professional nursing values, perceived work environment, and job satisfaction among critical care nurses in Palestine.

**Methods:**

This descriptive cross‐sectional study included 263 nurses working in critical care units across 15 hospitals in the West Bank, using a convenience sampling approach, with a response rate of 87.7%. Job satisfaction was treated as the primary dependent variable. Descriptive statistics, multiple linear regression, and mediation analyses were performed using SPSS 26.0.

**Results:**

Nurses reported strong professional values (116.25 ± 9.55), moderate perceptions of the work environment (2.42 ± 0.30) and moderate job satisfaction (89.17 ± 10.57). Work environment was the strongest predictor of job satisfaction (*β* = 0.576, *p* < 0.001), while workload (*β* = −0.166, *p* = 0.021) and intention to leave (*β* = −0.163, *p* < 0.001) were negative predictors; the model explained 47.4% of variance. Mediation analyses indicated significant indirect association between professional values and job satisfaction through work environment (*B* = 0.152), between the work environment and intention to leave through job satisfaction (*B* = −0.533), and between workload and intention to leave through job satisfaction (B = 0.082; All *p* < 0.001).

**Conclusion:**

Professional values were indirectly associated with job satisfaction through the perceived work environment. However, workload and intention to leave were associated with lower satisfaction. These findings highlight the need for adequately staffed, collaborative, well‐resourced critical care environments, including workload monitoring, shared decision‐making, supportive leadership and nurse recognition systems.

**Relevance to Clinical Practice:**

Nurse Managers should monitor workload, ensure adequate nurse–patient ratios, involve nurses in unit‐level decisions, provide recognition and strengthen interprofessional collaboration in critical care units.

**Patient or Public Contribution:**

No Patient or Public Contribution.

## 1 Introduction

Critical care nursing represents one of the most demanding and ethically sensitive areas of nursing practice because critical care nurses care for high‐acuity, critically ill patients whose conditions require intensive monitoring, complex interventions, technological therapies and ethical decision‐making (American Association of Critical‐Care Nurses [AACN] 2014). In Palestine, the health care system is affected by political instability, movement restrictions, emergencies, economic strain, fragmented services, workforce challenges and resource shortages. This context is crucial for examining factors shaping critical care nursing, particularly because nurses are expected to maintain ethical and professional standards while working under conditions that may restrict resources, autonomy, continuity of care and timely access to services (Aqtam and Shouli 2026).

In critical care units (CCUs), nurses provide continuous monitoring, manage patients receiving mechanical ventilation, administer and titrate complex medications, respond rapidly to clinical deterioration and coordinate care for patients who are often unable to speak for themselves. They also communicate with families during fear, uncertainty, suffering and difficult decisions (Davidson et al. 2017; Yoo et al. 2020). Therefore, critical care nursing requires both advanced clinical competence and strong professional values, including dignity, compassion, accountability, advocacy, confidentiality, justice, integrity and respect for human life (AACN 2014; American Nurses Association [ANA] 2025; International Council of Nurses [ICN] 2021; Ulrich 2025; Weis and Schank 2017).

Professional values are the beliefs, ethical standards, and principles that guide nurses' relationships with patients, families, colleagues, institutions and the profession. The International Council of Nurses (2021) emphasizes nursing responsibilities toward people, practice, the profession, global health and social justice. In CCU practice, values such as dignity, compassion, accountability, advocacy, confidentiality, justice, professional integrity and respect for human life are reflected in both major clinical decisions and daily care. These may include maintaining the privacy of sedated patients, communicating honestly with distressed family members, questioning unsafe practices and ensuring comfort when curative treatment is no longer possible (Weis and Schank 2017). Moral distress refers to the psychological discomfort nurses experience when they know the ethically appropriate action but are constrained from taking it because of organizational, resource, communication or authority‐related barriers (Aqtam et al. 2026; Morley et al. 2019). In CCUs, such distress may occur when nurses cannot provide the standard of care they believe patients deserve, especially when patient needs exceed available staff, equipment, time or institutional support.

However, professional values are not practiced in isolation. Nurses may believe in the value of advocacy, dignity, and safe care, but their ability to act according to these values depends greatly on the work environment (Ayed, Abu Ejheisheh, et al. 2024; Laari 2026). The nursing work environment refers to organizational and clinical conditions that support or limit professional nursing practice, including adequate staffing and resources, nurse participation in decision‐making, nursing foundations for quality care, managerial support, nurse–physician collaboration, teamwork, skilled communication, meaningful recognition and authentic leadership (AACN 2014; Lake 2002). Previous studies documented that favourable nursing work environments are associated with better nurse, patient and organizational commitment, quality of care, safety, satisfaction and workforce stability (Aiken et al. 2012; Emiralioğlu and Sönmez 2025; Havaei et al. 2022; Lake et al. 2019; Shajiri and Nahari 2026; Wei et al. 2018).

Conceptually, the current study is guided by a person‐environment fit perspective, which suggests that compatibility between individual values and workplace conditions is linked to more favourable work attitudes, including job satisfaction (Kristof‐Brown et al. 2005; Xiao et al. 2021). In this study, professional values represent nurses' ethical responsibilities and professional accountabilities, whereas the work environment represents the organizational context that enables or constrains professional nursing practice (ICN 2021; Lake 2002; Weis and Schank 2017). Job satisfaction reflects nurses' affective appraisal of their job and work experiences. From this perspective, congruence between nurses' professional values and supportive work conditions is expected to enhance job satisfaction. Conversely, incongruence between what nurses value and what the workplace allows may contribute to moral distress, frustration, dissatisfaction and reduced commitment to the workplace.

This relationship between professional values and the work environment is especially important in CCUs. Supportive work environments may help CCNs to translate their professional values into clinical practice. CCNs can better advocate for patients, maintain standards and provide compassionate care when they have adequate staffing levels, availability of resources, respectful communication and supportive leadership (AACN 2005; Abu Ejheisheh et al. 2025; Ayed, Ejheisheh, et al. 2024; Hiler et al. 2018; Leithe‐Lajord and Grønning 2025). In contrast, poor work environments may prevent nurses from delivering care according to their professional values. Nurses who value advocacy may feel powerless when their bedside concerns are ignored, while nurses who value dignity may experience moral strain when workload prevents basic comfort care (Hiler et al. 2018; Guttormson et al. 2022). Thus, professional values may increase job satisfaction when the work environment supports value‐based care; however, when conditions prevent them from delivering the care they deem appropriate, these same values may become a source of frustration (Ozdoba et al. 2024, 2025).

Job satisfaction is an important outcome in critical care nursing because it is linked to nurses' wellbeing, motivation, retention, quality of care, and patient safety (Lu et al. 2019; Zhao et al. 2025). Job satisfaction is shaped not only by individual attitudes but also by the structure and culture of the workplace (Körner et al. 2015; Kiptulon et al. 2024). For instance, in CCUs, job satisfaction is often challenged by high patient acuity, emotional pressure, exposure to death and suffering, heavy workloads, complex family interactions and constant vigilance (Dilig‐Ruiz et al. 2018; Quesada‐Puga et al. 2024). Previous studies show that autonomy, staffing, teamwork, stress, emotional exhaustion, leadership and professional support are important factors that influence job satisfaction among CCNs (Dilig‐Ruiz et al. 2018; Alzailai et al. 2021; Quesada‐Puga et al. 2024). Broader nursing literature also identifies workload, staffing shortages, long shifts, burnout, reward, control and managerial support as factors related to dissatisfaction and intention to leave (Dall'Ora et al. 2015, 2020; Lu et al. 2019; Bae 2024; Zhao et al. 2025).

Although professional values, work environment, ethical climate, burnout, moral distress and job satisfaction have been studied in nursing, many studies have focused on general hospital nurses, nursing students or nurse managers. These groups differ from CCNs, who work in environments marked by continuous high‐risk decision‐making, frequent ethical challenges, rapid patient deterioration and complex family needs (Alrashedi et al. 2025; Andersson et al. 2025; Grande et al. 2022). In Palestine, previous studies have mainly examined professional values, work environment and job satisfaction separately. For example, CCU nurses demonstrated strong professional values linked to caring behaviours, the CCU work environment was associated with professional quality of life and patient‐centred care, and job satisfaction was identified as a concern among CCNs in East Jerusalem (Abu Ejheisheh et al. 2025; Ayed, Abu Ejheisheh, et al. 2024; Bardhia et al. 2025; Darabee 2024). However, Palestinian studies have not examined professional values, perceived work environment and job satisfaction simultaneously among CCNs. Examining these variables together can clarify whether nurses' ethical commitments and workplace conditions are independently and jointly related to job satisfaction in a resource‐constrained critical care context.

Therefore, this study aimed to assess professional values, perceived work environment, and job satisfaction among CCNs in Palestine and to examine the interrelationships among these variables. The study addressed three research questions: (1) What are the levels of professional values, perceived work environment, and job satisfaction among Palestinian CCNs? (2) What are the relationships among professional values, perceived work environment and job satisfaction? (3) To what extent do professional values and perceived work environment predict job satisfaction among Palestinian CCNs? By focusing on Palestinian CCNs, this study addresses an important issue for nursing leadership, workforce sustainability and quality of critical care.

## 2 Methods

### 2.1 Study Design and Setting

This descriptive cross‐sectional study used a structured web‐based questionnaire administered between 1 March and 30 April 2026 across fifteen conveniently selected hospitals in the West Bank, Palestine. It focused on CCUs, including intensive care units, cardiac care units, neonatal intensive care units and paediatric intensive care units. This study was reported in accordance with the Strengthening the Reporting of Observational Studies in Epidemiology (Data S1).

### 2.2 Participants and Sampling

The target population was registered nurses working in CCUs. Eligibility criteria included registered nurses, currently working in a CCU, able to read Arabic and willing to participate. Nurses were excluded if they were working outside critical care, were student nurses or trainees, were on extended leave or submitted questionnaires with substantial missing data. Hospitals were selected based on CCU availability and administrative approval. A convenience sampling approach was used to recruit CCNs.

The sample size was estimated using Cochran's formula with finite population correction for an accessible population of approximately 350 CCNS, yielding a minimum of 184 at 95% confidence and 5% margin of error (*p* = 0.50). Although 350 CCNs were eligible, only 300 were accessible and invited because some were unavailable due to rotating shifts, leave, reassignment or limited access during data collection. Of 300 invited CCNs, 263 completed and returned the questionnaire, yielding a response rate of 87.7%. However, the use of convenience sampling may introduce selection bias, as CCNs who were available or willing to complete an online questionnaire may have been more likely to participate. Participating hospitals were selected based on CCU availability and administrative approval, and no formal comparison with non‐participating hospitals was possible; therefore, generalizability should be interpreted with caution.

### 2.3 Data Collection Instruments

Data were collected using a structured self‐administered Arabic questionnaire. The instruments were translated into Arabic by two bilingual nursing academics and independently back‐translated into English by another expert. A panel of five nursing faculty and CCNs reviewed the Arabic version for semantic, cultural and content equivalence. The final version was pilot‐tested with 30 CCNs to assess clarity, wording and completion time; these nurses were excluded from the final sample. The questionnaire included demographic and work‐related characteristics, professional nursing values, nursing practice environment and job satisfaction.

Demographic and work‐related variables included age, gender, marital status, education, hospital sector, job position, employment status, total nursing experience, critical care experience, type of CCU, shift pattern, nurse‐to‐patient ratio, number of night shifts per month, critical care training, ethics or professional values training, workload index and intention to leave. Intention to leave was coded as 1 = no, 2 = not sure, and 3 = yes. The workload index was developed for this study by combining three workload‐related indicators: nurse‐to‐patient ratio, number of night shifts per month, and shift pattern. Each component was coded so that higher values reflected greater workload burden, then summed/averaged to produce an overall workload score. Because this index was developed for this study and has not undergone psychometric validation, it was treated as an exploratory indicator. Findings involving this index should therefore be interpreted cautiously.

Professional nursing values were measured using the Nurses Professional Values Scale‐3 (NPVS‐3). The NPVS‐3 includes 28 items rated on a 5‐point Likert scale. The NPVS‐3 scores were calculated as total and mean scores. The possible total score ranges from 28 to 140, with higher scores indicating stronger professional nursing values. It includes three subscales: caring, activism, and professionalism.

The nursing practice environment was measured using the Practice Environment Scale of the Nursing Work Index (PES‐NWI). The PES‐NWI consists of 31 items rated on a 4‐point Likert scale. It includes five subscales: nurse participation in hospital affairs, nursing foundations for quality of care, nurse manager ability and support, staffing and resource adequacy and nurse‐physician relations. The PES‐NWI composite mean score was used as the main practice‐environment variable. Higher scores indicate a more favourable nursing practice environment. A commonly used interpretation is that mean scores above 2.50 indicate a favourable practice environment, whereas scores below 2.50 indicate a less favourable or unfavourable practice environment.

Job satisfaction was measured using the McCloskey/Mueller Satisfaction Scale (MMSS). The MMSS includes 31 items rated on a 5‐point Likert scale, assessing multiple dimensions of nurses' job satisfaction, including extrinsic rewards, scheduling, family‐work balance, co‐workers, interaction opportunities, professional opportunities, praise and recognition, and control and responsibility. The possible total score ranges from 31 to 155. Higher scores indicate greater job satisfaction. The MMSS global satisfaction mean score was used as the dependent variable.

The three instruments were used together to reflect the study framework: NPVS‐3 assessed nurses' professional values, PES‐NWI assessed the practice environment and MMSS assessed job satisfaction. This allowed examination of how individual professional values and organizational work conditions were associated with job satisfaction among CCNs. Internal consistency was good for the NPVS‐3 overall scale (*α* = 0.893), PES‐NWI overall scale (*α* = 0.868), and MMSS overall scale (*α* = 0.866).

### 2.4 Data Collection and Management

Data were collected electronically using Google Forms. The first page included the study information sheet and informed consent statement. Participants indicated consent by selecting the agreement option before completing the questionnaire. No names, employee numbers, phone numbers or other direct identifiers were collected. Participation was anonymous, and nurses could stop completing the questionnaire before submission. Data were exported to Microsoft Excel and IBM SPSS Statistics version 26. Files were stored in password‐protected locations accessible only to the research team. Data were screened for completeness, coding errors, out‐of‐range values and missing responses. Questionnaires missing more than 20% of items within a main scale were excluded. For minor missing data, scale scores were calculated when at least 80% of items in the relevant scale were completed; missing items were replaced using the participant's mean score for completed items on the same scale.

### 2.5 Data Analysis Plan

Data were analysed using IBM SPSS Statistics version 26. Frequencies and percentages were used for categorical variables. Means, standard deviations, and ranges were used for continuous variables. Internal consistency reliability was assessed using Cronbach's alpha for the NPVS‐3, PES‐NWI, and MMSS. Pearson correlation coefficients were used to examine bivariate associations between MMSS global satisfaction and professional values, practice environment, workload index, night shifts per month and intention to leave. Independent *t*‐tests and ANOVA were used to compare mean scores across selected groups. Multiple linear regression was conducted to identify factors associated with MMSS global job satisfaction. Predictors included age, gender, critical care training, ethics or professional values training, NPVS‐3 mean score, PES‐NWI composite score, workload index, night shifts per month and intention‐to‐leave score. Assumptions of regression were assessed using residual plots, normality tests, and homoscedasticity. Finally, mediation analyses were conducted using bootstrap procedures with 5000 resamples. Indirect effects were considered significant when the 95% confidence interval did not include zero. Statistical significance was set at *p* < 0.05, and all tests were two‐tailed.

### 2.6 Ethical Considerations

Ethical approval was obtained from the Institutional Review Board of Arab American University (approval number J‐2025/A/36/N). Administrative permission was obtained from participating hospitals. Participation was voluntary; informed consent was obtained electronically, and data were reported only in aggregate form. Unit managers were not involved in participant selection, consent, data collection or access to responses.

## 3 Results

### 3.1 Participant Characteristics

Among 263 CCNs, the mean age of the participants was 34.87 ± 7.18 years (range = 22–53). Slightly more than half of them were female (53.6%), and most held a bachelor's degree (*n* = 199, 75.7%). Their mean total nursing experience was 12.17 ± 7.25 years (range = 0.5–29.8), and their mean critical care experience was 7.27 ± 4.73 years. Most of participants worked in governmental hospitals (74.9%), 73.8% were staff nurses, and 81.7% were employed full time.

The most frequently reported CCU was the ICU (59.3%), followed by coronary care units (20.9%), NICUs (12.9%), and PICUs (6.9%). More than half of participants worked rotating shifts (51.7%). The most common nurse‐to‐patient ratio was 1:3 and more (*n* = 122, 46.4%). Most participants had received critical care training (70.0%), while 123 participants (46.8%) had received ethics or professional values training. One‐third of participants (30.8%) reported that they were considering leaving, while 79 (30.0%) were not considering leaving, and 103 (39.2%) were unsure (Table 1).

**TABLE 1:** Participant demographic and work‐related characteristics (*N* = 263).

Variable	*n* (%)
Gender	
Male	122 (46.4)
Female	141 (53.6)
Educational level	
Diploma	35 (13.3)
Bachelor's degree	199 (75.7)
Master's degree	29 (11.0)
Place of residence	
City	150 (57.0)
Village	96 (36.5)
Camp	17 (6.5)
Hospital sector	
Governmental	197 (74.9)
Private	66 (25.1)
Critical care unit	
ICU	156 (59.3)
Coronary care unit	55 (20.9)
NICU	34 (12.9)
PICU	18 (6.9)
Current job title	
Staff nurse	194 (73.8)
Charge nurse	42 (16.0)
Head nurse	20 (7.6)
Nurse manager	7 (2.7)
Employment status	
Full‐time	215 (81.7)
Part‐time	9 (3.4)
Contract	39 (14.8)
Shift pattern	
Morning	59 (22.4)
Evening	39 (14.8)
Night	29 (11.0)
Rotating	136 (51.7)
Weekly working hours	
< 36	16 (6.1)
36–40	120 (45.6)
41–48	100 (38.0)
> 48	27 (10.3)
Nurse‐to‐patient ratio	
1:1	33 (12.5)
1:2	108 (41.1)
1:3 or more patients per nurse	122 (46.4)
Critical care training	
Yes	184 (70.0)
No	79 (30.0)
Ethics/professional values training	
Yes	123 (46.8)
No	140 (53.2)
Considering leaving workplace	
Yes	81 (30.8)
No	79 (30.0)
Not sure	103 (39.2)

Abbreviations: ICU, intensive care unit; NICU, neonatal intensive care unit; PICU, paediatric intensive care unit.

### 3.2 Descriptive Statistics for Study Variables

Participants reported a mean of 3.69 ± 3.19 night shifts per month (range = 0–10). The mean workload index was 1.52 ± 0.63 (range = 0.25–3.18), and the mean leaving‐intention score was 2.01 ± 0.78 (range = 1–3).

The mean professional values total score was 116.25 ± 9.55 (range = 89–135). Among the NPVS‐3 subscales, caring had the highest mean score (4.45 ± 0.35), followed by professionalism (4.17 ± 0.39) and activism (3.84 ± 0.42).

The mean PES‐NWI composite mean score was 2.42 ± 0.30 (range = 1.62–3.21). Subscale means were highest for nursing foundations for quality of care (2.65 ± 0.34) and nurse‐physician relations (2.64 ± 0.43), whereas staffing and resource adequacy had the lowest mean score (1.97 ± 0.39).

The mean MMSS total score was 89.17 ± 10.57 (range = 56–112). Among MMSS dimensions, co‐workers had the highest mean score (3.33 ± 0.59), followed by interaction opportunities (3.18 ± 0.52) and praise and recognition (3.01 ± 0.40). Family‐work balance had the lowest mean score (2.63 ± 0.54), followed by extrinsic rewards (2.67 ± 0.47) and control and responsibility (2.76 ± 0.41) (Table 2).

**TABLE 2:** Descriptive statistics for continuous study variables.

Variable	M	SD	Range
Age, years	34.87	7.18	22–53
Total nursing experience, years	12.17	7.25	0.5–29.8
Critical care experience, years	7.27	4.73	0.2–21.5
Night shifts per month	3.69	3.19	0–10
Workload index	1.52	0.63	0.25–3.18
Leaving‐intention score	2.01	0.78	1–3
Nurses professional values total score	116.25	9.55	89–135
Caring subscale	4.45	0.35	3.30–5.00
Activism subscale	3.84	0.42	2.70–4.80
Professionalism subscale	4.17	0.39	2.75–5.00
Practice environment composite mean score	2.42	0.30	1.62–3.21
Nurse participation subscale	2.40	0.40	1.33–3.44
Nursing foundations subscale	2.65	0.34	1.50–3.40
Nurse manager support subscale	2.45	0.45	1.40–3.60
Staffing/resources subscale	1.97	0.39	1.00–3.00
Nurse‐physician relations subscale	2.64	0.43	1.67–3.67
Satisfaction Scale total	89.17	10.57	56–112
Extrinsic rewards subscale	2.67	0.47	1.33–4.00
Scheduling subscale	2.79	0.44	1.50–3.83
Family‐work balance subscale	2.63	0.54	1.00–4.00
Co‐workers subscale	3.33	0.59	1.50–5.00
Interaction opportunities subscale	3.18	0.52	1.50–4.25
Professional opportunities subscale	2.83	0.54	1.25–4.00
Praise and recognition subscale	3.01	0.40	2.00–4.00
Control and responsibility subscale	2.76	0.41	1.40–4.00

Abbreviations: MMSS, McCloskey/Mueller Satisfaction Scale; NPVS‐3, Nurses Professional Values Scale‐3; PES‐NWI, Practice Environment Scale of the Nursing Work Index.

### 3.3 Bivariate Associations

MMSS global satisfaction was positively correlated with professional values (*r* = 0.254, *p* < 0.001), and with the PES‐NWI composite score (*r* = 0.644, *p* < 0.001). Negative associations were observed between MMSS global satisfaction and workload index (*r* = −0.262, *p* < 0.001); night shifts per month (*r* = −0.150, *p* = 0.015); and leaving‐intention score (*r* = −0.293, *p* < 0.001). The PES‐NWI composite score was positively correlated with MMSS global satisfaction (*r* = 0.644, *p* < 0.001), and NPVS‐3 mean score was positively correlated with PES‐NWI composite score (*r* = 0.246, *p* < 0.001). Because age, total nursing experience, and critical‐care experience were highly intercorrelated (*p* < 0.01), only age was retained in the adjusted regression model to reduce redundancy and avoid instability in the estimates (Table 3).

**TABLE 3:** Means, standard deviations and Pearson correlation matrix for continuous variables (*n* = 263).

	1	2	3	4	5	6	7
1. Age	—						
2. Total nursing experience	0.966^a^	—					
3. Critical‐care experience	0.860^a^	0.884^a^	—				
4. Night shifts/month	−0.066	−0.061	−0.078	—			
5. Workload index	−0.102	−0.085	−0.099	0.752^a^	—		
6. NPVS	0.080	0.102	0.104	−0.087	−0.083	—	
7. PES	0.016	−0.002	−0.027	−0.161^a^	−0.215^a^	0.246^a^	—
8. MMSS	0.067	0.063	0.096	−0.150^b^	−0.262^a^	0.254^a^	0.644^a^

Abbreviations: MMSS, McCloskey/Mueller Satisfaction Scale; NPVS, Nurses Professional Values Scale‐3; PES, Practice Environment Scale of the Nursing Work Index.

^a^ *p* < 0.01.

^b^ *p* < 0.05.

### 3.4 Factors Associated With Job Satisfaction

Multiple linear regression was conducted to examine factors associated with MMSS global job satisfaction. The model included age, gender, critical care training, ethics or professional values training, NPVS‐3 mean score, PES‐NWI composite score, workload index, number of night shifts per month and leaving‐intention score. Assumption testing indicated no evidence of problematic multicollinearity, with variance inflation factors ranging from 1.02 to 2.47. Residual diagnostics supported the adequacy of the model, including normally distributed residuals, Shapiro–Wilk W = 0.995, *p* = 0.497, and no evidence of heteroscedasticity, Breusch–Pagan LM = 3.24, *p* = 0.954.

The regression model was statistically significant and explained 47.4% of the variance in MMSS global satisfaction (*R* ^2^ = 0.474, adjusted *R* ^2^ = 0.455, *F*(9, 253) = 25.31, *p* < 0.001). Higher MMSS global satisfaction was significantly associated with a more favourable practice environment (*B* = 0.645, *β* = 0.576, *p* < 0.001). Lower MMSS global satisfaction was significantly associated with higher workload index (*B* = −0.090, *β* = −0.166, *p* = 0.021) and stronger intention to leave the workplace (*B* = −0.071, *β* = −0.163, *p* < 0.001). Age, gender, critical care training, ethics or professional values training, professional values and number of night shifts per month were not significant adjusted predictors of MMSS global satisfaction (Table 4).

**TABLE 4:** Multiple linear regression of factors associated with MMSS global job satisfaction.

Predictor	*B*	SE	*β*	*t*	*p*	95% CI
Intercept	1.175	0.239	—	4.91	< 0.001	[0.703, 1.647]
Age	0.002	0.002	0.032	0.69	0.493	[−0.003, 0.006]
Gender, female	0.001	0.032	0.002	0.05	0.963	[−0.061, 0.064]
Critical care training, yes	−0.015	0.034	−0.021	−0.45	0.654	[−0.083, 0.052]
Ethics/professional values training, yes	0.030	0.032	0.043	0.91	0.364	[−0.034, 0.093]
Professional values, NPVS mean	0.078	0.049	0.078	1.60	0.112	[−0.018, 0.175]
Practice environment, PES composite	0.645	0.055	0.576	11.71	< 0.001	[0.537, 0.754]
Workload index	−0.090	0.039	−0.166	−2.32	0.021	[−0.166, −0.014]
Night shifts per month	0.010	0.007	0.094	1.35	0.178	[−0.005, 0.025]
Leaving‐intention score	−0.071	0.021	−0.163	−3.44	< 0.001	[−0.112, −0.030]

*Note:* Model fit: *R* ^2^ = 0.474, adjusted *R* ^2^ = 0.455, *F*(9, 253) = 25.31, *p* < 0.001. Gender was coded 0 = male and 1 = female. Critical care training and ethics/professional values training were coded 0 = no and 1 = yes. The leaving‐intention score was coded 1 = no, 2 = not sure, and 3 = yes.

Abbreviations: β, standardized coefficient; B, unstandardized coefficient; CI, confidence interval; SE, standard error.

### 3.5 Mediation Analyses

The mediation analyses identified three statistically significant indirect pathways. First, the practice environment partially mediated the association between professional nursing values and job satisfaction. The indirect effect was significant, *B* = 0.152, 95% CI [0.079, 0.225], *p* < 0.001. The direct effect also remained significant, *B* = 0.102, *p* = 0.037, indicating partial mediation. Overall, 59.8% of the association was mediated through the practice environment.

Second, job satisfaction significantly mediated the association between the practice environment and intention to leave. The indirect effect was significant and negative, *B* = −0.533, 95% CI [−0.759, −0.309], *p* < 0.001. After job satisfaction was included in the model, the direct effect of the practice environment on intention to leave was no longer significant, *B* = 0.118, *p* = 0.552. This finding suggests that the association between a more favourable practice environment and lower intention to leave operated primarily through higher job satisfaction.

Third, job satisfaction partially mediated the association between workload and intention to leave. The indirect effect was significant, *B* = 0.082, 95% CI [0.036, 0.137], *p* < 0.001, while the direct effect remained significant, *B* = 0.181, *p* = 0.017. Approximately 31.1% of the total association between workload and intention to leave was mediated through job satisfaction (Table 5). Finally, because the mediation analyses were based on cross‐sectional data, the indirect pathways should be interpreted as statistical associations and not as evidence of causal mediation or temporal ordering.

**TABLE 5:** Standardized total, direct and indirect effects for the mediation models (*n* = 263).

Path	Unstd.	Std.	SE	*t*‐value	*p*	*R* ^2^	LLCI	ULCI
**Model 1: PV ‐ > PE ‐ > JS**								
PV → PE	0.220	0.246	0.054	4.103	0.000***	0.061	0.114	0.325
PV → JS	0.102	0.102	0.049	2.102	0.037*	0.424	0.006	0.198
PE → JS	0.693	0.618	0.054	12.735	0.000***		0.586	0.800
*Indirect effect*								
PV → PE → JS	0.152	0.152	0.037				0.079	0.225
*Total effect*								
PV → JS	0.254	0.254	0.060	4.248	0.000***	0.065	0.136	0.372
**Model 2: PE ‐ > JS ‐ > LI**								
PE → JS	0.721	0.644	0.053	13.585	0.000***	0.414	0.617	0.826
PE → LI	0.118	0.046	0.199	0.596	0.552	0.087	−0.273	0.510
JS → LI	−0.739	−0.322	0.177	−4.165	0.000***		−1.089	−0.390
*Indirect effect*								
PE → JS → LI	−0.533	−0.208	0.113				−0.759	−0.309
*Total effect*								
PE → LI	−0.415	−0.161	0.157	−2.643	0.009**	0.026	−0.724	−0.106
**Model 3: WL ‐ > JS ‐ > LI**								
WL → JS	−0.141	−0.262	0.032	−4.388	0.000***	0.069	−0.205	−0.078
WL → LI	0.181	0.147	0.075	2.412	0.017*	0.106	0.033	0.329
JS → LI	−0.583	−0.254	0.139	−4.186	0.000***		−0.858	−0.309
*Indirect effect*								
WL → JS → LI	0.082	0.067	0.026				0.036	0.137
*Total effect*								
WL → LI	0.264	0.213	0.075	3.526	0.000***	0.045	0.116	0.411

*Note:* Indirect‐effect SEs and 95% CIs were estimated using 5000 bootstrap resamples.

Abbreviations: JS, job satisfaction; LI, leave intention; LLCI, lower limit confidence interval; PE, practice environment; PV, professional values; *R* ^2^, variance explained; SE, standard error; Std., standardized coefficients; ULCI, upper limit confidence interval; Unstd., unstandardized coefficients; WL, workload.

* *p* < 0.05.

** *p* < 0.01.

*** *p* < 0.001.

## 4 Discussion

This study assessed professional nursing values, perceived work environment and job satisfaction among CCNs in Palestine. The study findings provide three important contributions to critical care nursing knowledge. First, CCNs reported strong professional values, particularly in the caring domain, despite working in a resource‐constrained and politically complex health care context. Second, the practice environment was the strongest factor associated with job satisfaction, explaining more of the variance in satisfaction than professional values, workload, night shifts, training or demographic characteristics. Third, the mediation findings suggest that the practice environment is a key mechanism through which professional values are translated into job satisfaction, and that job satisfaction is an important pathway linking both the work environment and workload with intention to leave.

These findings are important because CCNs' professional commitment alone may not be sufficient to sustain satisfaction or retention. This interpretation is consistent with the study framework, in which professional values represent individual commitments, the practice environment represents organizational support, and job satisfaction reflects nurses' affective response to practicing within that context. Rather, CCNs appear to need work environments that enable them to enact their values in practice. In this study, professional values were positively correlated with job satisfaction, but they were not an independent predictor in the adjusted regression model. By contrast, the practice environment remained the strongest predictor of job satisfaction, suggesting that professional values may be necessary but not sufficient; CCNs may strongly value dignity, advocacy, compassion, and professional accountability, but satisfaction depends considerably on whether staffing, resources, leadership, teamwork and decision‐making structures allow these values to be expressed in daily care.

### 4.1 Professional Values and Job Satisfaction

The positive correlation between professional values and job satisfaction supports previous studies showing that professional values, ethical climate and job satisfaction are related among nurses (Ozdoba et al. 2024, 2025). Similarly, Eskandari Kootahi et al. (2023) found that professional values were associated with job satisfaction among neonatal intensive care nurses. However, in this study, professional values were no longer statistically significant after adjustment for the practice environment and workload. The loss of statistical significance for professional values in the adjusted model does not mean that values are unimportant. Rather, it suggests that professional values may influence satisfaction mainly when the work environment allows nurses to enact those values, which is consistent with person–environment fit theory and evidence linking professional values, ethical climate and job satisfaction among nurses (Aqtam, Shouli, Tumeh, and Ayed 2026; Kristof‐Brown et al. 2005; Eskandari Kootahi et al. 2023; Ozdoba et al. 2024, 2025). In unsupportive environments, strong values may increase frustration or moral distress rather than job satisfaction, particularly when nurses are unable to act according to their professional judgement because of workload, inadequate resources or organizational constraints (Aqtam, Shouli, Khaled, et al. 2026; Aqtam and Shouli 2026; Guttormson et al. 2022; Hiler et al. 2018). Therefore, professional values may contribute to satisfaction when supported by adequate staffing, resources, leadership, collaboration and opportunities for professional practice (AACN 2005; Wei et al. 2018; Emiralioğlu and Sönmez 2025).

This suggests that professional values may influence job satisfaction indirectly through supportive work conditions rather than acting as an independent predictor. This finding should not be interpreted as evidence that professional values are unimportant. Instead, it suggests that values require supportive organizational conditions to influence nurses' work experience. When nurses are unable to deliver the care they deem ethically necessary, strong values may increase moral tension rather than satisfaction. This tension can be understood as moral distress, particularly when nurses recognize the ethically appropriate action but are constrained by workload, limited resources or hierarchical decision‐making. In line with previous literature, CCNs often experience moral distress when they are unable to act according to their professional judgement because of organizational constraints, inadequate resources, hierarchical decision‐making, or perceived non‐beneficial treatment (Guttormson et al. 2022; Hiler et al. 2018). Therefore, professional values may become a source of satisfaction when the work environment supports ethical practice, but a source of frustration when CCNs are prevented from providing care consistent with those professional values. This distinction is particularly relevant in CCUs, where delays, omissions, or constraints in care can have immediate consequences for patients and their families.

### 4.2 The Central Role of the Work Environment

In the current study, the work environment was the strongest predictor of job satisfaction, indicating that CCNs who perceived their work environment more favourably reported higher job satisfaction. This may be because CCU nursing is highly dependent on staffing, resources, leadership support, communication and teamwork; therefore, organizational conditions may shape satisfaction more strongly than individual characteristics. This finding is in agreement with previous literature indicating that favourable nursing work environments are correlated with better nurse outcomes, including job satisfaction, organizational commitment, workforce stability and quality of care (Havaei et al. 2022; Pressley and Garside 2023; Shajiri and Nahari 2026; Wei et al. 2018). It is also consistent with ICU‐specific evidence from Palestine showing that the work environment is related to professional quality of life and is a primary predictor of patient‐centred care among nurses in intensive care units (Ayed, Ejheisheh, et al. 2024; Bardhia et al. 2025).

Compared with evidence from international and high‐income settings, where autonomy, professional support, leadership, staffing, shift patterns and work organization have been identified as important determinants of nurses' job satisfaction and retention, the stronger role of the work environment in this study may reflect the added influence of resource constraints, staffing shortages and system‐level instability in Palestinian CCUs (Aiken et al. 2012; Dall'Ora et al. 2015; Dilig‐Ruiz et al. 2018; Lu et al. 2019). In better‐resourced settings, professional development, autonomy, leadership support and career advancement may play a larger role in shaping job satisfaction (Dilig‐Ruiz et al. 2018; Lu et al. 2019; Wei et al. 2018). In contrast, in resource‐constrained Palestinian critical care settings, basic staffing adequacy, availability of supplies and equipment, supportive leadership, interprofessional collaboration and workload management may become more immediate determinants of satisfaction (Ayed, Abu Ejheisheh, et al. 2024; Bardhia et al. 2025; Darabee 2024). This may explain why the practice environment remained the strongest predictor of job satisfaction, whereas professional values were not independently significant after adjustment.

The nurse work environment is particularly important in CCUs, where patient care is highly interdependent, time‐sensitive, and technologically complex. In such settings, CCNs depend on adequate staffing, functioning equipment, reliable supplies, supportive leadership, clear communication and effective interprofessional collaboration to deliver safe and high‐quality care (American Association of Critical‐Care Nurses [AACN] 2005; Dilig‐Ruiz et al. 2018; Wei et al. 2018; Ayed, Ejheisheh, et al. 2024; Bardhia et al. 2025). Similarly, the AACN identifies skilled communication, true collaboration, effective decision‐making, appropriate staffing, meaningful recognition and authentic leadership as essential standards for establishing and sustaining healthy critical care work environments (AACN 2005). The findings of the current study reinforce the relevance of these standards in the Palestinian CCU context (Ayed, Abu Ejheisheh, et al. 2024) and support broader evidence linking positive nursing work environments with job satisfaction, care quality and nurse retention (Dilig‐Ruiz et al. 2018; Wei et al. 2018; Ayed, Ejheisheh, et al. 2024).

In this study, staffing and resource adequacy had the lowest work environment score. This is a particularly important finding for critical care nursing. Nearly half of participants reported a nurse‐to‐patient ratio of 1:3 or more, which may be difficult to reconcile with the intensity of monitoring, medication titration, ventilator care, infection prevention, family support and rapid response required in CCUs (Elmdni 2025; Ernest et al. 2025). Inadequate staffing and resources may reduce CCNs' ability to provide timely, safe and compassionate care, thereby lowering job satisfaction and increasing psychological strain. Previous research has similarly identified staffing, workload, mandatory overtime and work hours as important determinants of job satisfaction, burnout and intention to leave among nurses (Bae 2024; Dall'Ora et al. 2015; Elmdni 2025; Zhao et al. 2025).

### 4.3 Workload, Shifts and Satisfaction

The study findings revealed that workload was negatively associated with job satisfaction and remained a significant predictor in the adjusted regression model. This finding is clinically meaningful because CCU nurses work in high‐acuity environments requiring constant vigilance, rapid decision‐making and emotional engagement with critically ill patients and families. This finding aligns with prior evidence showing that job satisfaction among CCNs is strongly influenced by workload, staffing adequacy, stress, leadership, teamwork, autonomy and emotional exhaustion (Aqtam et al. 2023; Dilig‐Ruiz et al. 2018; Quesada‐Puga et al. 2024). It is also consistent with broader nursing literature showing that heavy workload and organizational stressors contribute to dissatisfaction and turnover intention (Lu et al. 2019; Zhao et al. 2025).

Although night shifts were negatively correlated with job satisfaction, this relationship was not significant in the adjusted regression model. This suggests that dissatisfaction related to night work may be partly explained by broader workload and environmental conditions rather than night duty alone. Night shifts may be particularly challenging when staffing is limited, managerial support is less available, patient acuity is high and nurses have reduced access to multidisciplinary resources. This interpretation is consistent with evidence showing that night shift, work hours, shift length and workload interact with organizational conditions to influence nurses' satisfaction, burnout and intention to leave (Alanmi et al. 2025; Aqtam et al. 2023; Bae 2024; Dall'Ora et al. 2015).

In this study, the lowest satisfaction domains were family–work balance, extrinsic rewards, and control and responsibility. These findings indicate that dissatisfaction appears to be influenced less by interpersonal relationships and more by structural aspects of employment. In contrast, co‐workers and opportunities for interaction received the highest satisfaction scores, suggesting that peer support and teamwork continue to serve as important strengths in Palestinian CCUs. Team relationships may help nurses manage the emotional and clinical intensity of CCU work. Previous evidence has shown that high workloads, interpersonal relationships, teamwork, and organizational culture shape CCNs' stress and well‐being; stronger teamwork is also associated with job satisfaction and lower burnout or turnover intentions (Bragadóttir et al. 2023; Kiptulon et al. 2024; Körner et al. 2015; Tümer et al. 2025; Zaghini et al. 2024). Therefore, nurse leaders should preserve these relational strengths while addressing structural sources of dissatisfaction.

### 4.4 Mediation Findings and Contribution to Knowledge

The mediation analyses add a novel contribution by identifying potential pathways linking professional values, the work environment, job satisfaction, workload, and intention to leave. However, because mediation analysis was based on cross‐sectional data, these pathways should be interpreted as statistical associations rather than evidence of causal mediation. Prior studies have shown associations between nurses' professional values and job satisfaction, as well as between ethical/professional climate and job satisfaction (Eskandari Kootahi et al. 2023; Ozdoba et al. 2024, 2025). The work environment is also consistently identified as an important determinant of nurses' job satisfaction, quality of work life, burnout and retention, particularly in critical care contexts (Dilig‐Ruiz et al. 2018; Lu et al. 2019; Ayed, Abu Ejheisheh, et al. 2024; Quesada‐Puga et al. 2024; Zhao et al. 2025). First, the work environment partially mediated the relationship between professional values and job satisfaction. This suggests that nurses with stronger professional values may be more satisfied when their work environment supports professional nursing practice. In practical terms, professional values are more likely to enhance satisfaction when CCNs have adequate resources, supportive managers, collaborative relationships, and opportunities to participate in decisions affecting patient care (AACN 2005; Wei et al. 2018; Emiralioğlu and Sönmez 2025).

Second, job satisfaction fully mediated the relationship between the work environment and intention to leave, emphasizing a critical workforce insight. It suggests that a favourable work environment may reduce intention to leave primarily by improving CCNs' satisfaction with their work. This pathway aligns with previous studies showing that positive work environments are associated with higher job satisfaction, organizational commitment and workforce stability (Shajiri and Nahari 2026; Wei et al. 2018; Zhao et al. 2025). In CCUs, retaining experienced CCNs is particularly important, as expertise develops over time through exposure to complex patient care, advanced technologies, ethical decision‐making and interdisciplinary coordination (AACN 2014; Davidson et al. 2017; Hiler et al. 2018; Tümer et al. 2025).

Third, job satisfaction partially mediated the relationship between workload and intention to leave. This indicates that workload may increase intention to leave both directly and indirectly through reduced satisfaction. The direct effect may reflect fatigue, stress, physical demands and perceived unsustainable working conditions, while the indirect effect suggests that workload also influences retention by shaping CCNs' overall evaluation of their job. This finding is aligned with previous literature that staffing level, shift length and mandatory overtime are associated with CCNs' job satisfaction (Dall'Ora et al. 2015; Bae 2024). In critical care settings, workload‐related stress, burnout and occupational fatigue are especially relevant because CCNs work in high‐acuity environments that require continuous vigilance and complex decision‐making (Guttormson et al. 2022; Quesada‐Puga et al. 2024; Alanmi et al. 2025). Therefore, this finding reinforces the importance of workload management as both a staff wellbeing strategy and a retention strategy (Pressley and Garside 2023; Zhao et al. 2025).

### 4.5 Implications for Critical Care Nursing Practice and Leadership

The study findings have direct and important implications for CCU leadership, hospital administration, and workforce policy. First, improving the practice environment should be considered a strategic priority for CCNs' satisfaction and retention. Practical strategies may include shared governance councils, structured mentorship, resilience and peer‐support programmes, leadership coaching, staff recognition systems, ethics rounds, and regular workload review. Consistent with healthy work environment standards for critical care nursing, targeted interventions should emphasize adequate staffing, resource availability, supportive nurse management, meaningful recognition, shared governance and effective nurse‐physician collaboration (AACN 2005). These interventions may help support positive nursing environments and therefore improve the quality and safety of care (Havaei et al. 2022; Wei et al. 2018).

Second, nurse managers should prioritize workload and staffing adequacy by using acuity‐based staffing models rather than relying only on fixed nurse‐to‐patient ratios. In CCUs, staffing allocation should consider patient instability, vasoactive medication use, mechanical ventilation, isolation requirements, family support needs, admissions, discharges and end‐of‐life care. When safe staffing is compromised, workload monitoring should be routine, visible and linked to clear escalation mechanisms.

Third, the study findings revealed that ethics or professional values training was associated with higher professional values but was not independently linked to job satisfaction, suggesting that education alone is insufficient. Therefore, professional values education should be integrated with organizational support. Ethics education, moral case deliberation, communication training and reflective practice with leadership structures that empower CCNs to raise concerns, participate in decisions and advocate for patients without fear of dismissal or retaliation.

Fourth, nurse managers should prioritize the lowest scored domains, particularly family‐work balance, extrinsic rewards, and control and responsibility to improve CCNs' satisfaction. Practical strategies include fair and flexible scheduling, transparent distribution of night shifts, adequate rest, recognition of CCU emotional labour, professional development opportunities and greater CCN involvement in unit‐level decisions. Finally, job satisfaction, as a mediator between work environment and turnover intention, should be monitored as a workforce indicator and early warning of instability. Hospitals should regularly assess CCNs' satisfaction, workload and turnover intention to inform staffing, leadership, and retention strategies. At the unit level, nurse managers should implement shared governance, fair scheduling, structured mentorship, staff recognition systems and regular workload review. At the hospital level, administrators should invest in staffing adequacy, leadership development, psychological support and reliable access to essential resources. At the policy level, decision‐makers should develop context‐sensitive critical care staffing standards and retention strategies for CCNs working in resource‐constrained Palestinian hospitals.

### 4.6 Implications for the Palestinian Health Care Context

The Palestinian context gives these findings particular importance. CCNs practice within a health system affected by political instability, movement restrictions, fragmented services, workforce shortages, economic pressure, and resource constraints. These conditions may intensify workload and limit the availability of equipment, medications, supplies, functioning infrastructure and continuing professional development (Aqtam and Shouli 2026). Despite these challenges, nurses in the current study reported strong professional values, indicating a high level of professional commitment.

However, the study findings also caution against relying on CCNs' commitment as a substitute for organizational support. Resilience and professional values should not be used to normalize unsafe staffing, inadequate resources, or unsustainable workloads. A resilience‐informed approach should therefore emphasize both individual coping and organizational responsibility, including psychological safety, supportive leadership, adequate staffing and opportunities for recovery after stressful clinical events. In resource‐constrained settings, investment in CCU nursing work environments is both a workforce priority and a patient safety priority. Supporting CCNs to practice according to their values may be especially important in contexts where critical care services are under pressure and experienced CCU nurses are difficult to replace.

### 4.7 Strengths and Limitations

This study has several strengths. It focused specifically on CCNs, a group whose work differs substantially from general hospital nursing because of the intensity, complexity, and ethical sensitivity of CCU care. The study also examined professional values, the practice environment, job satisfaction and intention to leave together, rather than treating them as isolated variables.

However, several limitations should be considered when interpreting study findings. First, the cross‐sectional design prevents causal inference. Although the mediation analyses identified statistically significant indirect pathways, these findings should be interpreted as associations rather than evidence of causal mediation because temporal ordering among variables could not be established. Longitudinal studies are needed to determine whether changes in the practice environment lead to subsequent improvements in job satisfaction and reductions in intention to leave. Second, convenience sampling was used, and hospitals were selected based on the presence of CCUs and administrative approval. Therefore, the findings may not be generalizable to all CCNs in Palestine. CCNs who responded may also have differed from non‐respondents in motivation, satisfaction, workload, or professional engagement. Third, data were collected using self‐report questionnaires. This may introduce social desirability bias, particularly in relation to professional values, where CCNs may be inclined to report high endorsement of ethical and professional standards. Self‐reported satisfaction, workload, and intention to leave may also be influenced by recent workplace events or temporary emotional states. Finally, because data were collected only from participating West Bank hospitals, the findings may not be transferable to Gaza, East Jerusalem, or non‐participating hospitals where staffing patterns, emergency burden, infrastructure, and access to supplies may differ substantially. Future research should include broader geographical representation where feasible.

### 4.8 Recommendations for Future Research

Future studies should use longitudinal designs to examine whether improvements in staffing, leadership, collaboration, and resource adequacy lead to sustained improvements in job satisfaction and retention. Intervention studies are also needed to test CCU‐specific strategies such as acuity‐based staffing, shared governance, structured debriefing, ethics rounds, nurse‐led quality improvement and peer support programmes. Qualitative research would provide deeper insight into how Palestinian CCNs experience professional values, moral distress, workload, satisfaction, and decisions about leaving. Mixed‐methods studies may be particularly useful for explaining the gap between strong professional values and moderate satisfaction.

Future research should also examine patient and organizational outcomes. Linking practice environment measures with missed nursing care, adverse events, infection prevention, family satisfaction, nurse turnover and patient safety indicators would strengthen the evidence base for investing in critical care nursing work environments.

## 5 Conclusion

Critical care nurses in participating West Bank hospitals reported high levels of professional values but only moderate job satisfaction and perceptions of their practice environment. The practice environment was the strongest predictor of job satisfaction, while higher workload and stronger intention to leave were associated with lower satisfaction. Professional values were positively related to job satisfaction, but this relationship was partly explained by the practice environment, suggesting that values contribute to satisfaction when nurses are supported to enact them in practice. Job satisfaction also mediated the relationships between the practice environment, workload and intention to leave, highlighting its central role in CCU workforce sustainability.

These findings indicate that sustaining critical care nursing requires more than strengthening individual professional values. It requires healthy, adequately staffed, well‐resourced and supportive work environments that enable nurses to provide the ethical, safe and compassionate care they value. In resource‐constrained and politically complex health care contexts, investment in CCU practice environments is essential for nurse wellbeing, retention and patient safety.

## Author Contributions

**Bahaaeddin M. Hammad:** conceptualization, investigation, writing – original draft, methodology, validation, writing – review and editing, formal analysis, project administration, data curation, supervision, visualization, resources.

## Funding

The author has nothing to report.

## Disclosure

The author declares that the work described in this manuscript has not been published previously, except in the form of a preprint, abstract, published lecture, academic thesis, or registered report. The manuscript is not under consideration for publication elsewhere. The publication of this article has been approved by the author and by the responsible authorities at the institution where the work was carried out. If accepted, the article will not be published elsewhere in the same form, in English or in any other language, including electronically, without the written consent of the copyright holder.

## Ethics Statement

Ethical approval was obtained from the Institutional Review Board of Arab American University, approval number R‐2026/A/92/N. Administrative permission was obtained from the participating hospitals. Participation was voluntary, informed consent was obtained electronically and data were reported only in aggregate form. Unit managers were not involved in participant selection, consent, data collection or access to responses.

## Consent

The author has nothing to report.

## Conflicts of Interest

The author declares no conflicts of interest.

## Supporting information


**Data S1:** Supporting Information.

## Data Availability

The data that support the findings of this study are available from the corresponding author upon reasonable request.
